# Chemokine/Cytokine Levels Correlate with Organ Involvement in PR3-ANCA-Associated Vasculitis

**DOI:** 10.3390/jcm10122715

**Published:** 2021-06-19

**Authors:** Janina Müller-Deile, Christian Jaremenko, Hermann Haller, Mario Schiffer, Marion Haubitz, Silke Christiansen, Christine Falk, Lena Schiffer

**Affiliations:** 1 Department of Nephrology and Hypertension, Friedrich-Alexander-University (FAU) Erlangen-Nuremberg, 91054 Erlangen, Germany; mario.schiffer@uk-erlangen.de; 2 Institute for Nanotechnology and Correlative Microscopy eV, INAM, 91301 Forchheim, Germany; christian.jaremenko@inam-forchheim.de (C.J.); silke.christiansen@mpl.mpg.de (S.C.); 3 Institute of Optics, Information and Photonics, Friedrich-Alexander-University (FAU) Erlangen-Nuremberg, 91054 Erlangen, Germany; 4 Department of Nephrology, Hannover Medical School, 30625 Hannover, Germany; haller.hermann@mh-hannover.de; 5 Department of Nephrology and Hypertension, Center for Internal Medicine and Medical Clinic III, Klinikum Fulda, 36043 Fulda, Germany; Marion.Haubitz@Klinikum-Fulda.de; 6 Institute of Transplant Immunology, Hannover Medical School, 30625 Hannover, Germany; Falk.Christine@mh-hannover.de; 7 Department of Pediatric Nephrology, Hannover Medical School, 30625 Hannover, Germany; schiffer.lena@mh-hannover.de

**Keywords:** ANCA-associated vasculitis, cytokine profile, biomarker, kidney involvement

## Abstract

Background: ANCA-associated vasculitis (AAV) is a rare small vessel disease characterized by multi-organ involvement. Biomarkers that can measure specific organ involvement are missing. Here, we ask whether certain circulating cytokines and chemokines correlate with renal involvement and if distinct cytokine/chemokine patterns can differentiate between renal, ear/nose/throat, joints, and lung involvement of AAV. Methods: Thirty-two sets of Birmingham vasculitis activity score (BVAS), PR3-ANCA titers, laboratory marker, and different cytokines were obtained from 17 different patients with AAV. BVAS, PR3-ANCA titers, laboratory marker, and cytokine concentrations were correlated to different organ involvements in active AAV. Results: Among patients with active PR3-AAV (BVAS > 0) and kidney involvement we found significant higher concentrations of chemokine ligand (CCL)-1, interleukin (IL)-6, IL21, IL23, IL-28A, IL33, monocyte chemoattractant protein 2 (MCP2), stem cell factor (SCF), thymic stromal lymphopoietin (TSLP), and thrombopoietin (TPO) compared to patients without PR3-ANCA-associated glomerulonephritis. Patients with ear, nose, and throat involvement expressed higher concentrations of MCP2 and of the (C-X-C motif) ligand-12 (CXCL-12) compared to patients with active AAV and no involvement of these organs. Conclusion: We identified distinct cytokine patterns for renal manifestation and for ear, nose and throat involvement of PR3-AAV. Distinct plasma cytokines might be used as non-invasive biomarkers of organ involvement in AAV.

## 1. Introduction

Antineutrophil cytoplasmic antibodies (ANCA) associated vasculitis is a relatively rare and potential life threatening multi systemic autoimmune disease [1]. Organ involvement includes ear, nose, and throat (ENT); lung; heart; kidney; bowel; skin; nerves; and joints [2]. The serological presence of ANCA that recognize the neutrophil cytoplasmic antigens proteinase 3 (PR3) and myeloperoxidase (MPO) is the defining serological characteristic of all ANCA-associated vasculitides [3,4]. Entities of AAV include granulomatosis with polyangiitis (GPA; formerly known as Wegener’s Granulomatosis), microscopic polyangiitis (MPA), and eosinophilic granulomatosis with polyangiitis (EGPA; formerly known as Churg–Strauss Syndrome) [5]. Absolute ANCA titers correlate only roughly with severity of disease, and they do not distinguish between organ involvements. Their predictive value as a biomarker for relapses is a matter of debate as rising titers have been reported in up to 40% of patients without relapse. Moreover, sensitivity of an increasing ANCA titer for diagnosis of relapse ranged from 24% to 100% [6,7]. Thus, there is limited use to serial ANCA measurements to guide treatment decisions for individual patients with AAV [8].

However, the pathogenic role of antineutrophil cytoplasmic antibodies in ANCA-associated glomerulonephritis is highlighted in several studies and well accepted [9].

Renal involvement is a frequent finding in patients with AAV and is the most common cause of progressive glomerulonephritis [10]. Interestingly, passive transfer of purified MPO-ANCA or splenocytes from MPO-deficient mice immunized with purified MPO into wild type mice induced necrotizing pauci-immune glomerulonephritis [11,12]. Serum creatinine at diagnosis, sclerotic lesions, and the number of normal glomeruli at kidney biopsy are unspecific but so far the best predictors of renal outcome [13]. If untreated renal involvement is an important cause of end-stage renal disease (ESRD) in general. Moreover, renal manifestation is associated with an increased morbidity and patients with AAV with glomerulonephritis have a significant lower 10-year survival rate compared to patients with no renal involvement. Thus, it is of great clinical relevance to diagnose and to treat glomerular involvement of the disease early [2]. So far, renal biopsy is still the gold standard to establish the diagnosis and severity of glomerulonephritis in AAV. However, as an invasive method it bears complications and cannot regularly be performed during follow up visits [14]. Furthermore, kidney biopsy may be biased by sampling error. Therefore, noninvasive or less-invasive biomarkers for renal involvement of AAV are clinical useful. Ideally, these biomarkers could be not only used for diagnosis, but also to detect relapses and to guide therapy in a patient centered approach.

Experimental and clinical data suggest that pathogenesis in (ANCA)-associated vasculitis is driven by ANCA-mediated production of proinflammatory cytokines by neutrophils and monocytes [15]. A variety of cytokines and chemokines are upregulated in AAV, however serum levels do not correlate with serum levels of ANCA [16,17]. Despite it has been found that cytokine profiles differ in PR3- and MPO-AAV [18]. ANCA have been shown to induce interleukin (IL)-1-beta, IL6, and IL8. IL10 was also suggested as a potential marker of early systemic AAV [19].

However, a systematic approach to identify cytokine and chemokine profiles for prediction of specific organ involvement for management of AAV is still missing.

The principal goal of our study was to identify cytokine/chemokine patterns that better detect organ-specific disease involvement in PR3-AAV. Using cytokine arrays, we measured multiple cytokines in patients’ plasma including different interleukins, CC-chemokine ligands, chemokine (C-X-C motif) ligands, monocyte chemoattractant proteins, thymic stromal lymphopoietin (TSLP), thrombopoietin (TPO), and stem cell factors (SCFs) and correlated them to ANCA titer, laboratory markers, disease activity score, and organ involvement.

## 2. Materials and Methods

### 2.1. Study Population

Paired plasma samples and clinical parameters drawn at disease onset and at 11–13 months follow up were obtained from 14 patients with active PR3-AAV. Birmingham vasculitis activity score (BVAS), laboratory marker, and plasma cytokine levels were documented at both visits for all patients. Furthermore, clinical data and blood samples were obtained from 3 more patients at active disease stage. In total 32 different samples were measured. The patients were recruited at Hannover Medical School. Disease activity was classified according to the BVAS. Remission was defined as BVAS = 0 and active disease as BVAS > 0. Patients with systemic infections were excluded. Patients’ demographics are shown in Table 1.

The study was conducted in accordance with the Declaration of Helsinki and was approved by the Institutional Review Board of Hannover Medical School. Written informed consent was obtained from all participants. Peripheral blood was collected in pyrogen-free tubes containing ethylenediaminetetraacetic acid as anticoagulant (Sarstedt, Nümbrecht, Germany). Tubes were immediately inversed and centrifuged at 2500× *g* for 10 min, and plasma samples were stored at −80 °C.

### 2.2. Cytokine Array

A MILLIPLEX MAP Human Cytokine/Chemokine Magnetic Bead Panel II-Immunology Multiplex Assay was used to measure 22 human cytokines in 31 different plasma samples of patients with AAV. The assay allowed the simultaneously analysis of multiple cytokine and chemokine biomarkers with bead-based and luminex technology. The array was performed according to the manufacture’s instruction (HCYTOMAG-60K Millipore, Merck KGaA, Darmstadt, Germany).

### 2.3. Assessment of Organ Involvement of the Disease

Medical history and physical examination were used to calculate ear/nose/throat and joint activity. Kidney involvement was defined by kidney biopsy showing ANCA-associated glomerulonephritis.

### 2.4. Statistics

The distributions of the individual cytokines were analyzed using the appropriate parametric or nonparametric analyses to determine whether the differences between the respective populations were significant. Whenever possible, a two-sample and two-sided independent t-test [20] was performed for this purpose, which compares the means of the two populations. The fundamental requirement, an approximate Gaussian distribution, as well as the homoscedasticity was tested for each cytokine individually using the Shapiro–Wilk [21] and the Levene test [22], respectively. Welch’s *t*-test [23] was employed in the case of different variances but approximate Gaussian distribution, whereas the non-parametric Mann–Whitney U test procedure [24] was applied in the case of non-Gaussian distribution. For the cytokine distributions with significant differences, additional boxes and whisker plots are presented to simplify the visualization of the distributions and to highlight the median, the upper and lower quartile, the whiskers with 1.5 of the interquartile distances, as well as the outliers.

## 3. Results

### 3.1. Patients’ Characteristics

Seventeen patients with PR3-AAV were included in the study. Eight of these were female and nine were male. We have initial clinical, laboratory, and cytokine level of all these patients and additional clinical, laboratory, and cytokine level on 15 of these patients 1 year later. For two patients, follow up blood samples were not available. In total, 32 different blood samples and clinical scores were used for the analysis. For 15 patients paired samples were available that were taken approximately 1 year apart when the patients were in partial or complete remission as well as two additional single samples of ANCA positive patients at initial presentation with active disease. Therefore, 23 patient samples were taken in active disease and 9 patient samples were taken in complete remission. The mean age of patients was 54.1 ± 13.0. A PR3-ANCA titer at active disease stage was 331.4 ± 596.2 and 141.3 ± 314.5 at remission. BVAS was 8.5 ± 6.4 at active disease stage and 0.0 ± 0.0 at time of remission (Table 1). Patients’ comorbidities, main organ involvement and medication at time of initial presentation and follow up are also given in Table 1.

### 3.2. Correlations of Plasma Cytokines and Disease Activity

We correlated 32 PR3-ANCA titers, laboratory parameters, and cytokines with disease activity measured by BVAS. BVAS was significantly higher in active disease compared to inactive disease stage (*p* < 0.001) and PR3-ANCA titer or CRP were not significantly different between active phase of the disease and remission (Table 2 and supplementary Figure S1). None of the cytokines measured correlated with PR3-ANCA titer of the patients.

### 3.3. Correlations of Plasma Cytokines and Ear/Nose/Throat Activity

**Figure 1:**
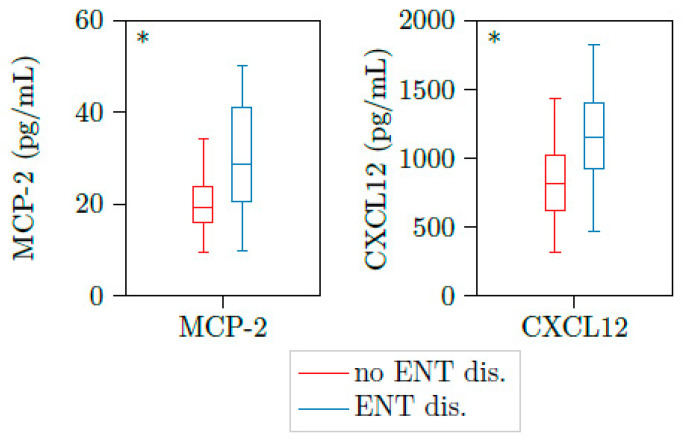
MCP2 and CXCL12 are upregulated in patients with active AAV and ENT involvement. Illustration of MCP2 and CXCL12 plasma concentrations of 23 samples with active PR3-AAV. Both cytokines were significantly elevated in patients with ear, nose and throat involvement (*n* = 11) versus those without these organ’s involvement (*n* = 10). * *p* < 0.05, boxes and whisker plots present distribution of the median, the upper, and lower quartile, the whiskers with 1.5 of the interquartile distances, as well as the outliers.

Ear, nose, and throat (ENT) and lung activity are the most frequent findings in active PR3-AAV. From 23 samples with active PR3-AAV (BVAS > 0), BVAS, PR3-ANCA titer and CRP were not able to differentiate between patients with ENT involvement and patients without these organ involvements. Of note, the chemokine (C-C motif) ligand 8 (CCL8), also known as monocyte chemoattractant protein 2 (MCP2), and the cytokine CXCL12 were significantly elevated in disease stages with ENT activity versus no activity (Table 3 and Figure 1).

### 3.4. Correlations of Plasma Cytokines and Kidney Involvement

Next, we correlated BVAS and laboratory parameters of samples with active PR3-AAV (BVAS > 0) to the subgroup of patients with active vasculitis and kidney involvement. In patients of active PR3-AAV with renal involvement, mean PR3-ANCA titer was 246.86 (range: 0.00–1024.00) at active disease and 368.38 (range: 0.00–2048.00) at the time of remission. BVAS was higher in active PR3-AAV with glomerulonephritis (12.86; range: 8.00–19.00) compared to those with active PR3-AAV but no glomerulonephritis (6.62; range: 1.00–26.00).

**Figure 2:**
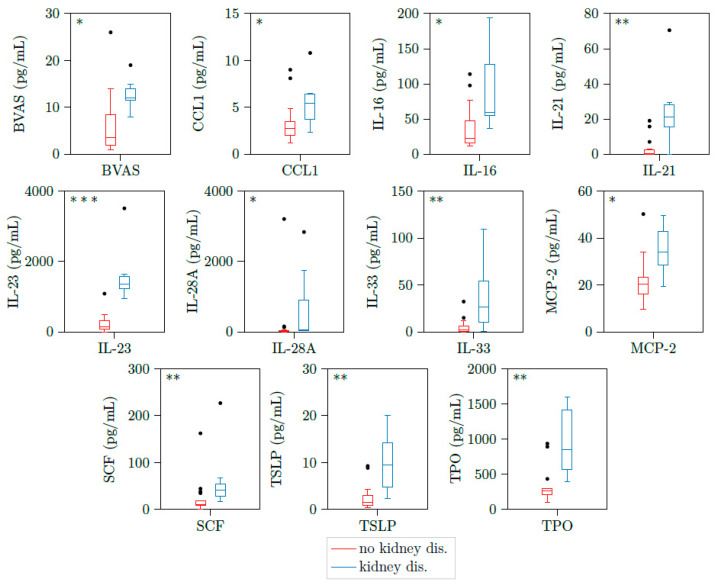
CCL1, IL16, IL21, IL23, IL28A, IL33, MCP2, SCF, TSLP, and TPO are upregulated in patients with active ANCA-associated vasculitis and kidney involvement. Illustration of BVAS, CCL1, IL16, IL21, IL23, IL28A, IL33, MCP2, CSF, TSLP, and TPO plasma concentrations of 23 samples with active PR3-AAV. All cytokines were significantly elevated in patients with kidney involvement (*n* = 7) versus those without kidney involvement (*n* = 16). * *p* < 0.05; ** *p* < 0.01, *** *p* < 0.001, boxes and whisker plots present distribution of the median, the upper and lower quartile, the whiskers with 1.5 of the interquartile distances, as well as the outliers.

We could detect significant higher concentrations of CCL1, IL16, IL21, IL23, IL28A, IL33, MCP2, SCF, TSLP, and TPO in patients with kidney involvement compared to active PR3-AAV patients without glomerulonephritis (Table 4 and Figure 2).

**Figure 3:**
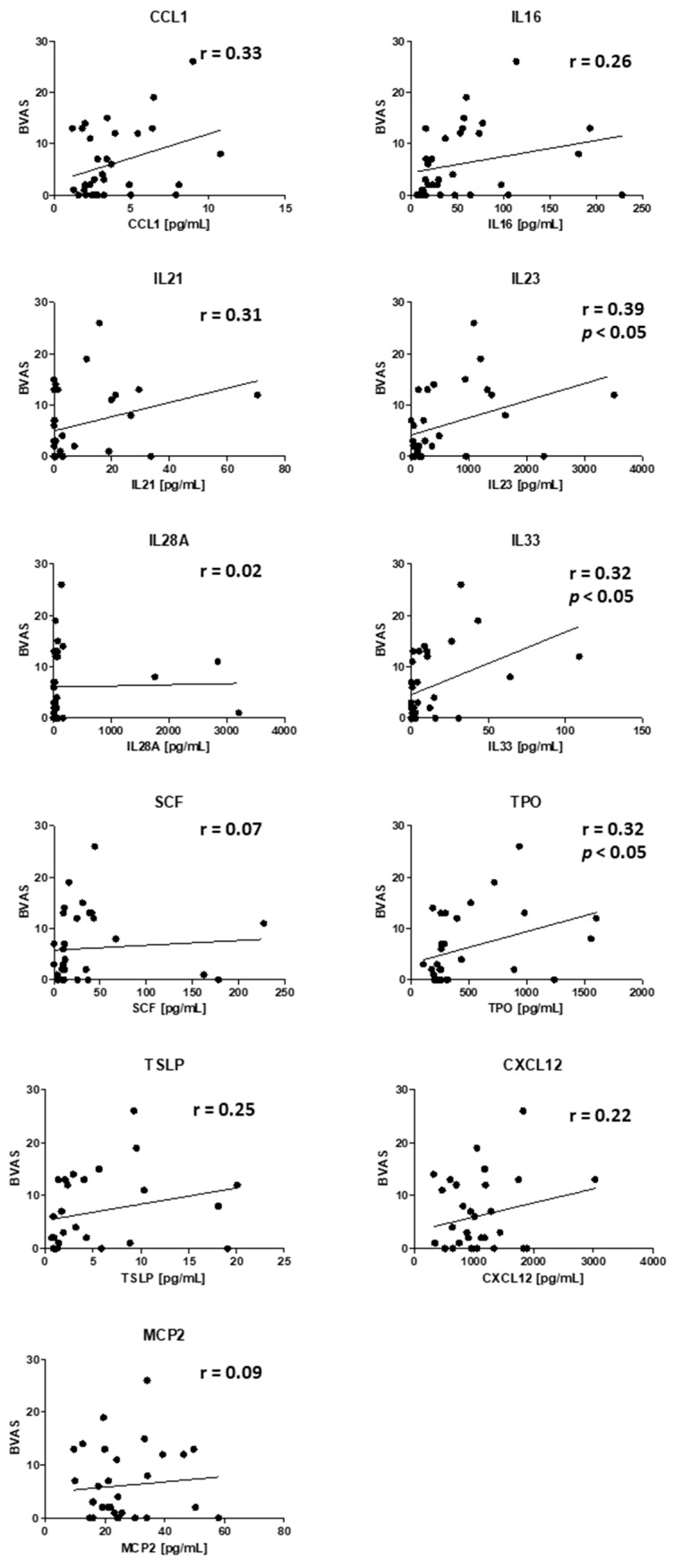
Correlation of BVAS and cytokines upregulated in active PR3-AAV. r = correlation coefficient, *p* < 0.05 = significant correlation, *n* = 32 samples.

Next, we looked for correlation of BVAS and cytokines upregulated in active PR3-AAV with kidney or ENT involvement. CCL1, IL6, IL-21, IL-23. IL-33, TPO, TSLP, and CXCL12 positively correlated with BVAS. This correlation was significant for IL-23, IL-33, and TPO (Figure 3).

### 3.5. Correlations of Plasma Cytokines and Joint and Lung Activity

**Figure 4:**
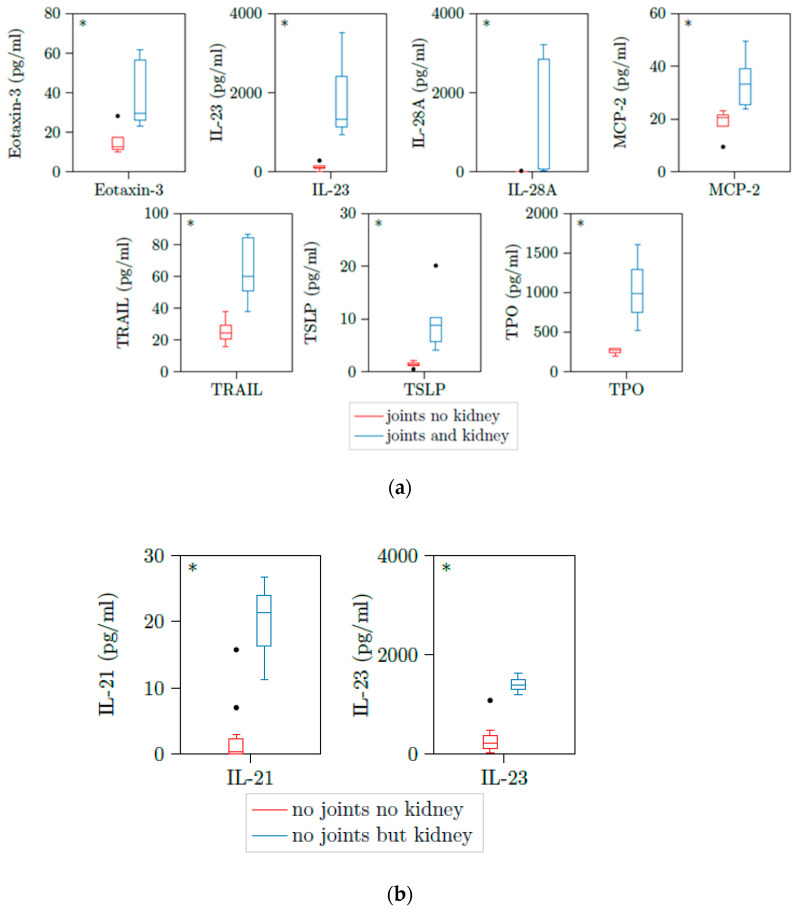
(**a**) Eotaxin-3, IL-23, IL-28A, MCP2, TRAIL, TSLP, and TPO are upregulated in patients with active AAV with both joint involvement and kidney involvement. Illustration of Eotaxin3, IL-23, IL-28A, MCP2, TRAIL, TSLP, and TPO plasma concentrations of 13 samples with active PR3-AAV and joint involvement. All cytokines were significantly elevated in patients with additional kidney involvement versus those without kidney involvement. * *p* < 0.05; boxes and whisker plots present distribution of the median, the upper and lower quartile, the whiskers with 1.5 of the interquartile distances, as well as the outliers. (**b**) IL-21 and IL-23 are upregulated in patients with active AAV with no joint involvement but kidney involvement. Illustration of IL-21 and IL-23 plasma concentrations of 9 samples with active PR3-AAV and joint involvement. All cytokines were significantly elevated in patients with additional kidney involvement versus those without kidney involvement. * *p* < 0.05; boxes and whisker plots present distribution of the median, the upper and lower quartile, the whiskers with 1.5 of the interquartile distances, as well as the outliers.

We could not see any correlation between cytokine levels and joint involvement or lung involvement of patients with active PR3-AAV (Supplementary Tables S1 and S2). However, when we further differentiated into patients with and without renal involvement in these groups, we could see statistically differences in some cytokines. When we compared patients with joint involvement and kidney involvement versus joint involvement but no kidney involvement, we found significant elevation of Eotaxin3 and TRAIL next to IL-23, IL-28A, MCP2, TSLP, and TPO seen in patients with kidney involvement before (Table 4 and Table 5 and Figure 4a). Patients with no joint but kidney involvement had significant elevated levels of IL-21 and IL-23 versus patients with neither joint involvement nor kidney involvement (Table 5 and Figure 4b). Therefore, IL-23 was a significant marker for kidney involvement independent of joint involvement.

**Figure 5:**
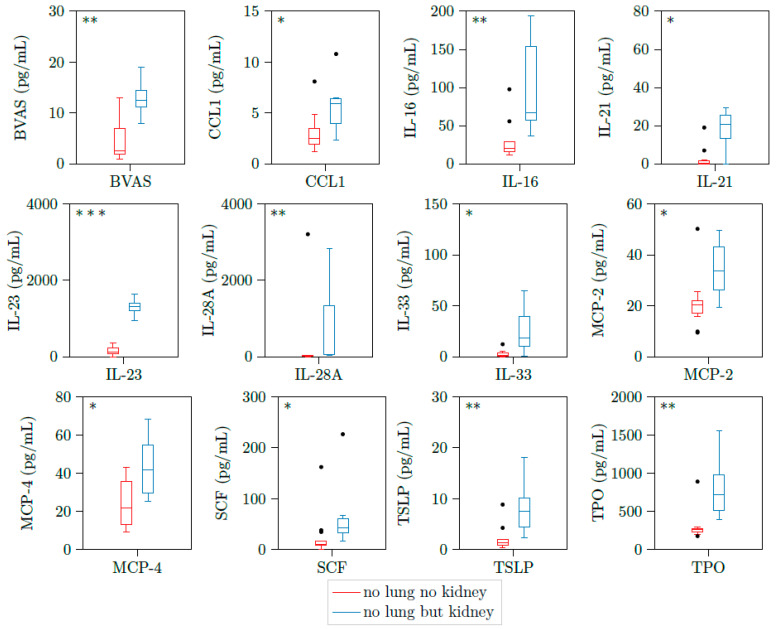
BVAS, CCL1, IL-16, IL-21, IL-23, IL-28A, IL-33, MCP2, MCP4, SCF, TSLP, and TPO are upregulated in patients with active AAV with both lung involvement and kidney involvement. Illustration of BVAS, CCL1, IL-16, IL-21, IL-23, IL28-A, IL-33, MCP2, MCP4, SCF, TSLP, and TPO plasma concentrations of 18 samples with active PR3-AAV and joint involvement. All cytokines were significantly elevated in patients with additional kidney involvement versus those without kidney involvement. * *p* < 0.05; ** *p* < 0.01, *** *p* < 0.001, boxes and whisker plots present distribution of the median, the upper and lower quartile, the whiskers with 1.5 of the interquartile distances, as well as the outliers.

When we compared patients with lung involvement and kidney involvement to patients with lung involvement but no kidney involvement we identified an additional cytokine which was MCP4 that was significant elevated in patients with both organ involvements (Table 6 and Figure 5). Therefore, we identified distinct cytokine profiles for constellations of different organ involvements in AAV.

## 4. Discussion

AAV is a rare and potential life-threatening multi-systemic autoimmune disease. Organ involvement can include kidney, ENT, heart, lung bowel, skin, nerves, and joints [2]. Renal involvement is frequently present in PR3-AAV and is, if untreated, an important cause of end-stage renal disease (ESRD) that is associated with a poor patient outcome and prognosis. As morbidity and mortality is significantly higher in AAV with glomerulonephritis compared to no renal involvement, it is important to diagnose and treat glomerular involvement of the disease early [2]. Different studies have shown that some chemokines and cytokines are elevated in active AAV, but a systematic approach regarding chemokine/cytokine levels and correlation with organ manifestations is missing so far.

We hypothesize that plasma chemokines/cytokines might be upregulated in different clinical and even subclinical presentations of the disease and specific patterns might be useful in the future to screen for specific organ involvement in AAV. We measured a variety of chemokine/cytokine concentrations in plasma of patients with PR3-AAV and correlated them to organ involvement, disease activity, and ANCA titers. We were also able to perform a serial measurement of all parameters at two different time points one year apart in most of our patients.

Well in line with previous studies, we found that the elevation of the inflammatory marker C-reactive protein (CRP) did not function as a predictor of relapse or organ manifestation [25]. ANCA-titers are a helpful tool for the diagnosis of AAV. However, besides the importance for the diagnosis of AAV it has to be considered that some patients with clinically and pathologically established disease stay seronegative, and some patients tend to stay ANCA positive after remission. Moreover, the course of ANCA titer does not always correlate with organ manifestation and disease activity, questioning the use of serial ANCA measurements in clinical routine. A meta-analysis by *Tomasson* et al. with nine studies found that rising ANCA or persistently positive ANCA titers were poorly associated with flares [8]. Methodological heterogeneity between laboratories combined with shortcomings with respect to internal validity further have questioned the use of ANCA titers for monitoring the follow-up of patients [6].

Differences in circulating cytokines were more strongly associated with kidney involvement of AAV than ANCA titers or BVAS. Similar to our findings, a low or even inverse correlation of cytokines with ANCA titers has been previously described [26,27].

Unfortunately, a reliable evaluation of the cytokine profile according to each of the BVAS-items is not possible due to the small sample size of our cohort. However, a significant positive correlation was seen for BVAS and IL-23, for BVAS and IL-33, and for BVAS and TPO. It is tempting to speculate that cytokines can detect subclinical disease activity better and therefore could serve as novel biomarkers that can detect disease activity earlier than BVAS; this hypothesis has to be tested in future studies.

In our study, cohort kidney and ENT involvement were the only organ involvements that correlated with significant elevation in specific cytokine levels suggesting heterogeneity in PR3-AAV subtypes especially associated with kidney involvement.

In particular, we found that CCL1, IL-16, IL-21, IL-23, IL-28A, IL-33, MCP2, CSF, TSLP, and TPO were elevated in PR3-AAV with kidney involvement. Well in line with our findings, several members of the CC chemokine family including CCL1 (TCA3) have been previously described in an experimental model of crescentric glomerulonephritis [28]. Activated endothelial cells, macrophages, and T lymphocytes secrete CCL1. It has been found in kidney transplant rejection but so far, no association with human AAV was described [29]. Different interleukins were shown to modulate NF-κB pathways. For example, IL-11 treatment decreases glomerular nuclear factor “kappa-light-chain-enhancer” (NF-kappa) activity and reduces renal injury in experimental glomerulonephritis [30]. IL-6 cytokine family members can play either a deleterious or a protective role in response to kidney disease. Signaling pathways induced by IL-6 cytokine family members include signal transducers and activators of transcription (STAT), mitogen-activated protein kinase (MAPK) and phosphoinositide 3-kinase (PI3K) [31]. Immune modulation with IL-4 and IL-10 prevented crescent formation and glomerular injury in experimental glomerulonephritis. On the other hand, B cell-derived IL-4 acts on podocytes to induce proteinuria and foot process effacement most likely by activating STAT6 [32]. The data suggest that a fine-tuned cytokine interplay is required for proper glomerular function.

IL-16 is a T cell chemoattractant produced by peripheral mononuclear cells. It has been recently reported as a predictor for acute kidney injury and as a biomarker for disease activity in systemic sclerosis with skin involvement [33,34]. However, to the best of our knowledge, an association with AAV has never been described. Of note, *Hladinova* et al. found increased levels of soluble ST2 in patients with active ANCA-associated vasculitis. This is of special interest since soluble ST2 is known to be the receptor of IL33 that was statistically elevated in our cohort [35]. Well in line with our finding, *Hoffmann* et al. found elevated serum IL-33 concentrations in AAV-patients compared to healthy controls, but no significant difference between active and inactive disease [27]. Moreover, we found a significant elevation of the interleukins IL-21 and IL-23 in patients with ANCA-associated glomerulonephritis. Previous studies described elevated IL-21 producing Th-cells in patients with AAV and found elevated IL-23 levels in active AAV compared to controls, however, in difference to our study, specific organ involvement was not examined [36].

MCP2 (CCL8) is an interferon-regulated gene, which has been previously implicated in other autoimmune diseases and in human and rodent crescentic GN [16,37]. However, we are the first to describe a correlation to human kidney involvement in AAV. Both, stem cell factor (SCF) and thymic stromal lymphopoietin (TSLP), are linked to the pathogenesis of renal diseases. SCF is known as a potential driver of tissue inflammation and fibrosis in human crescentric glomerulonephritis [38,39]. Thymic stromal lymphopoietin (TSLP) is a cytokine involved in T cell maturation by activation of antigen presenting cells and B-cell lymphopoiesis. Overexpression in mice results in an immune complex driven, aggravated membranoproliferative glomerulonephritis involving several glomerular cell types [40,41].

Interestingly, platelet to lymphocyte ratio was associated with the current activity of AAV [42]. This reactive thrombocytosis is induced by chronic inflammation which accelerates TPO production as well as production of cytokines which regulate megakaryocyte differentiation and proliferation. In line with this, TPO was another factor that was significantly increased in AAV with renal involvement compared to no renal involvement in our cohort. We did not find any significant correlations of chemokine and cytokine profiles regarding the involvement of the lung or joints; however, we discovered two cytokines (MCP2 and CXL12) that were elevated in ENT involvement. While MCP2 levels were also increased in renal involvement, CXCL12 was exclusively elevated in our patients with ENT involvement. The CXCL12 chemokine axis can chemotactically accumulate inflammatory cells to local tissues and regulate the release of inflammatory factors. In AAV, increased CXCL12 levels have been previously described in the sputum of ANCA+ patients [43]. Well in line with our finding, *Radjewski* et al. described CXCL12 as a biomarker whose levels can be increased in chronic inflammation of the paranasal tissue in general [44]. Many chemokines and cytokines have been implicated in leukocyte recruitment into inflamed tissues in autoimmune diseases, thus representing promising targets for therapeutic interventions [45]. Targeted therapies against chemokines and cytokines are under development and some have already been used in pre-clinical and clinical studies for the treatment of autoimmune inflammatory diseases [46,47]. It is tempting to speculate that the identified chemokines/cytokines in our cohort could represent novel targets in AAV with organ involvement.

Taken together, we could identify in our cohort the activation of distinct systemic chemokine/cytokine signatures that correlated with renal involvement in active AAV, but not with other disease manifestations. One cytokine was exclusively upregulated in ENT involvement.

Our study has limitations due to the small number of patients included. However, using a multiplex approach we were able to measure 22 different cytokine concentrations from each sample simultaneously. Furthermore, we additionally performed serial measurements in the same patients, correlated our results to different organ involvements, disease activity score, and established laboratory markers. Interestingly, all of the identified chemokines/cytokines in our cohort are already linked to renal diseases in rodent models and/or in patients. However, their pathogenic role in disease manifestation of AAV is not well understood and deserves further investigations.

## 5. Conclusions

Our study demonstrated a distinct and promising cytokine profile that was able to differentiate active kidney involvement of ANCA-associated vasculitis and one cytokine exclusively elevated in patients with ENT involvement. Moreover, our results may represent future biomarkers or might even serve as targets for therapeutic approaches in AAV patients. Further prospective studies are needed to confirm our findings.

## Figures and Tables

**Table 1:** Patients’ demographics.

Patient Demographics	
**Age; *n* = 17**	**mean ± std.**
age	54.1 ± 13.0
**Gender**	***n***
f/m	8/9
**PR3-ANCA titer**	**mean ± std.**
active disease	331.4 ± 598.2
remission	141.3 ± 314.5
**BVAS**	**mean ± std.**
active disease	8.5 ± 6.4
remission	0.0 ± 0.0
**Comorbidities; *n* = 17**	***n* yes/no (% yes/no)**
hypertension	11/6 (64.7/35.3)
diabetes	2/15 (11.8/88.2)
cardio vascular disease	0/17 (0/100)
malignancies	0/17 (0/100)
kidney transplantation	0/17 (0/100)
dialysis	2/15 (11.8/88.2)
**Main organ involvement at initial presentation; *n* = 17**	***n* yes/no (% yes/no)**
renal	7/10 (41.2/58.8)
ear, nose and throat	10/7 (58.8/41.2)
joint	7/10 (41.2/58.8)
lung	5/12 (29.4/70.6)
central nerve system	6/11 (35.3/64.7)
**Main organ involvement at follow up; *n* = 15**	***n* yes/no (% yes/no)**
renal	0/15 (0.0/100.0)
ear, nose and throat	2/13 (13.3/86.7)
joint	7/10 2/13 (13.3/86.7)
lung	0/15 (0.0/100.0)
central nerve system	2/13 (13.3/86.7)
**Medication at initial presentation; *n* = 17**	***n* yes/no (% yes/no)**
Steroids	13/4 (76.5/23.5)
Cyclophosphamide	3/14 (17.6/82.4)
Azathioprine	5/12 (29.4/70.6)
Mycophenolate mofetil	3/14 (17.6/82.4)
Methotrexate	2/15 (11.8/88.2)
Cyclosporine	0/17 (0/100)
Rituximab	0/17 (0/100)
Plasmapheresis	0/17 (0/100)
**Medication at follow up; *n* = 15**	***n* yes/no (% yes/no)**
Steroids	11/4 (73.0/27.0)
Cyclophosphamide	0/15 (0.0/100.0)
Azathioprine	2/13 (13.3/82.7)
Mycophenolate mofetil	2/13 (13.3/82.7)
Methotrexate	1/14 (6.7/93.3)
Cyclosporine	0/15 (0/100)
Rituximab	0/15 (0/100)
Plasmapheresis	0/15 (0/100)

PR3: proteinase 3 positive, ANCA: Antineutrophil cytoplasmic antibody, BVAS: Birmingham vasculitis activity score.

**Table 2:** Summary of all tested cytokines, laboratory parameter, PR3-ANCA titer, and BVAS in active AAV and in remission.

Cytokine	Active Mean (min–max); *n* = 23	Remission Mean (min–max); *n* = 9	* = *p* < 0.05; *** = *p* < 0.001; n.s. = not significant
ANCA	331.39 (0.00−2048.00)	141.33 (0.00−1024.00)	n.s.
BVAS	8.52 (1.00−26.00)	0.00 (0.00−0.00)	***
CCL1	4.02 (1.19−10.80)	3.37 (1.57−7.91)	n.s.
CCL15	2712.58 (322.19−5778.18)	2764.14 (1252.99−6326.59)	n.s.
CCL17	50.60 (0.00−123.75)	59.96 (7.03−125.37)	n.s.
CCL21	55.15 (0.00−256.08)	42.15 (6.42 v 157.79)	n.s.
CCL27	369.37 (17.98−757.08)	413.92 (148.57−1152.58)	n.s.
CRP	15.93 (0.30−145.00)	1.81 (1.00−3.00)	n.s.
CXCL12	1063.73 (317.83−3026.04)	1071.81 (511.06−1878.92)	n.s.
CXCL13	22.10 (1.89−97.34)	13.17 (3.22−29.82)	n.s.
CXCL5	507.91 (53.44−1315.25)	552.03 (50.23−1290.88)	n.s.
Eotaxin-2	423.54 (85.43−655.17)	456.92 (174.48−735.23)	n.s.
Eotaxin-3	40.93 (7.39−195.33)	40.55 (18.40−152.34)	n.s.
IL-16	54.65 (11.47−193.47)	57.99 (6.75−228.25)	n.s.
IL-20	48.95 (0.78−254.13)	42.65 (13.16−153.65)	n.s.
IL-21	9.97 (0.00−70.53)	4.17 (0.00−33.62)	n.s.
IL-23	650.77 (0.00−3511.30)	431.55 (26.03−2295.86)	n.s.
IL-28A	371.64 (0.00−3208.29)	36.91 (0.00−158.17)	n.s.
IL-33	15.44 (0.00−109.11)	6.07 (0.00−30.86)	n.s.
MCP-2	25.54 (9.47−50.23)	26.82 (14.72−58.03)	n.s.
MCP-4	32.15 (7.87−100.94)	30.06 (9.29−84.76)	n.s.
SCF	35.99 (0.00−226.84)	30.96 (3.85−178.01)	n.s.
TPO	514.48 (105.91−1602.86)	361.96 (199.93−1238.14)	n.s.
TRAIL	60.49 (10.34−176.74)	72.63 (16.45−238.06)	n.s.
TSLP	4.83 (0.36−20.09)	3.40 (0.44−19.08)	n.s.
creatinine	178.00 (33.00-802.00)	113.50 (74.00−282.00)	n.s.
hemoglobin	13.19 (11.00−15.80)	12.85 (11.10−14.20)	n.s.
leucocytes	8.53 (2.00−17.40)	5.70 (4.30−7.40)	*
thrombocytes	245.05 (13.00−591.00)	228.62 (75.00−341.00)	n.s.

AAV: ANCA associated vasculitis, CCL: C-C Motif Chemokine Ligand 2, CXCL: -X-C Motif Chemokine Ligand 1, IL: interleukin, MCP: monocyte chemoattractant protein 2, SCF: stem cell factors, TPO: thrombopoietin, TRAIL: Tumor Necrosis Factor Related Apoptosis Inducing Ligand, TSLP: thymic stromal lymphopoietin.

**Table 3:** Cytokines, laboratory parameter, PR3-ANCA titer and BVAS at active AAV differentiated in presence of ear, nose, and throat (ENT) involvement and no ENT involvement.

Cytokine	Active (ENT Affected) Mean (min–max); *n* = 12	Inactive (ENT Not Affected) Mean (min–max); *n* = 11	* = *p* < 0.05; n.s. = not significant
ANCA	392.17 (2.00−2048.00)	265.09 (0.00−2048.00)	n.s.
BVAS	10.17 (2.00−26.00)	6.73 (1.00−19.00)	n.s.
CCL1	4.50 (1.84−9.01)	3.50 (1.19−10.80)	n.s.
CCL15	2921.78 (322.19−4627.90)	2484.36 (349.69−5778.18)	n.s.
CCL17	57.56 (0.00−123.75)	43.01 (15.50−94.13)	n.s.
CCL21	58.58 (5.03−198.89)	51.42 (0.00−256.08)	n.s.
CCL27	429.99 (17.98−757.08)	303.23 (51.00−482.23)	n.s.
CRP	13.93 (1.00−32.00)	18.13 (0.30−145.00)	n.s.
CXCL12	1289.93 (466.15−3026.04)	816.95 (317.83−1434.82)	*
CXCL13	19.63 (1.89−83.24)	24.80 (3.73−97.34)	n.s.
CXCL5	539.80 (187.10−1315.25)	473.13 (53.44−1067.55)	n.s.
Eotaxin-2	475.61 (211.65−655.17)	366.74 (85.43−538.32)	n.s.
Eotaxin-3	45.06 (10.21−120.52)	36.42 (7.39−195.33)	n.s.
IL-16	60.17 (15.89−193.47)	48.63 (11.47−181.22)	n.s.
IL-20	52.25 (1.92−153.98)	45.35 (0.78−254.13)	n.s.
IL-21	13.70 (0.00−70.53)	5.90 (0.00−26.68)	n.s.
IL-23	830.31 (0.00−3511.30)	453.28 (32.49−1629.89)	n.s.
IL-28A	271.77 (0.00−2838.47)	480.58 (0.00−3208.29)	n.s.
IL-33	17.42 (0.00−109.11)	13.28 (0.00−64.37)	n.s.
MCP-2	30.62 (9.89−50.23)	20.00 (9.47−34.15)	*
MCP-4	39.28 (12.66−100.94)	24.37 (7.87−50.07)	n.s.
SCF	40.69 (0.03−226.84)	30.87 (0.00−162.36)	n.s.
TPO	604.01 (249.50−1602.86)	416.00 (105.91−1556.27)	n.s.
TRAIL	68.12 (15.70−176.74)	52.16 (10.34−134.17)	n.s.
TSLP	5.08 (0.46−20.09)	4.55 (0.36−18.09)	n.s.
creatinine	183.11 (33.00−802.00)	173.40 (63.00−662.00)	n.s.
hemoglobin	13.30 (11.30−15.80)	13.06 (11.00−15.10)	n.s.
leucocytes	9.14 (4.00−17.20)	7.86 (2.00−17.40)	n.s.
thrombocytes	247.27 (166.00−348.00)	242.60 (13.00−591.00)	n.s.

**Table 4:** Cytokines, laboratory parameter, PR3-ANCA titer, and BVAS at active AAV with and without kidney involvement.

Cytokine	Active (Kidney Affected) Mean (min–max); *n* = 7	Inactive (Kidney Not Affected) Mean (min–max); *n* = 16	* = *p* < 0.05; ** = *p* < 0.01; *** = *p* < 0.001; n.s. = not significant
ANCA	246.86 (0.00−1024.00)	368.38 (0.00−2048.00)	n.s.
BVAS	12.86 (8.00−19.00)	6.62 (1.00−26.00)	*
CCL1	5.55 (2.35−10.80)	3.35 (1.19−9.01)	*
CCL15	3456.04 (322.19−5778.18)	2387.32 (349.69−3909.46)	n.s.
CCL17	63.32 (0.00−123.75)	45.03 (15.50−101.86)	n.s.
CCL21	75.05 (5.03−256.08)	46.45 (0.00−198.89)	n.s.
CCL27	398.45 (17.98−757.08)	356.64 (51.00−719.80)	n.s.
CRP	14.53 (2.00−32.00)	16.49 (0.30−145.00)	n.s.
CXCL12	1202.39 (466.15−3026.04)	1003.06 (317.83−1823.04)	n.s.
CXCL13	31.09 (3.39−97.34)	18.17 (1.89−83.24)	n.s.
CXCL5	434.64 (252.89−653.00)	539.97 (53.44−1315.25)	n.s.
Eotaxin-2	463.00 (148.80−655.17)	406.28 (85.43−579.55)	n.s.
Eotaxin-3	62.83 (26.21−195.33)	31.34 (7.39−120.52)	n.s.
IL-16	93.63 (36.87−193.47)	37.59 (11.47−113.97)	*
IL-20	71.76 (1.92−254.13)	38.97 (0.78−153.98)	n.s.
IL-21	25.61 (0.00−70.53)	3.12 (0.00−19.06)	**
IL-23	1666.17 (936.37−3511.30)	244.61 (0.00−1086.40)	***
IL-28A	691.58 (28.11−2838.47)	231.66 (0.0−3208.29)	*
IL-33	37.87 (0.86−109.11)	5.62 (0.00−32.29)	**
MCP-2	35.13 (19.40−49.68)	21.34 (9.47−50.23)	*
MCP-4	40.06 (18.87−68.22)	28.69 (7.87−100.94)	n.s.
SCF	64.33 (16.12−226.84)	23.60 (0.00−162.36)	**
TPO	962.79 (397.49−1602.86)	335.15 (105.91−936.12)	**
TRAIL	64.21 (10.34−134.17)	58.86 (15.70−176.74)	n.s.
TSLP	10.00 (2.31−20.09)	2.56 (0.36−9.27)	**
creatinine	294.50 (90.00−802.00)	146.93 (33.00−662.00)	n.s.
hemoglobin	13.59 (11.30−15.80)	12.99 (11.00−15.00)	n.s.
leucocytes	10.67 (2.00−17.20)	7.46 (4.00−17.40)	n.s.
thrombocytes	277.00 (13.00−591.00)	229.07 (148.00−320.00)	n.s.

**Table 5:** (left) Cytokines, laboratory parameter, PR3-ANCA titer, and BVAS at active AAV with joint involvement differentiated into patients with and without kidney involvement. (right) Cytokines, laboratory parameter, PR3-ANCA titer, and BVAS at active AAV with no joint involvement differentiated into patients with and without kidney involvement.

Cytokine	Active (Joints No Kidney) Mean (min–max); *n* = 4	Inactive (Joints and Kidney) Mean (min–max); *n* = 5	* = *p* < 0.05; n.s. = not significant	Active (No Joints No Kidney) Mean (min–max); *n* = 10	Inactive (No Joints But Kidney) Mean (min–max); *n* = 3	* = *p* < 0.05; n.s. = not significant
ANCA	534.00 (8.00–2048.00)	332.80 (128.00−1024.00)	n.s.	324.60 (0.00−2048.00)	106.67 (0.00−256.00)	n.s.
BVAS	8.50 (1.00–13.00)	10.40 (1.00−15.00)	n.s.	6.50 (2.00−26.00)	13.00 (8.00−19.00)	n.s.
CCL1	1.94 (1.19–3.43)	3.62 (1.96−6.39)	n.s.	4.02 (2.01−9.01)	7.57 (5.44−10.80)	n.s.
CCL15	2976.61 (2568.80–3315.45)	2289.54 (322.19−4627.90)	n.s.	2327.43 (641.91−3909.46)	4364.76 (2905.70−5778.18)	n.s.
CCL17	26.20 (15.50–55.75)	50.11 (0.00−111.88)	n.s.	52.83 (23.63−101.86)	80.42 (46.15−123.75)	n.s.
CCL21	17.33 (0.00–34.27)	48.58 (5.03−97.60)	n.s.	62.04 (19.52−198.89)	106.35 (22.95−256.08)	n.s.
CCL27	342.61 (271.61–418.81)	311.34 (17.98−757.08)	n.s.	380.26 (148.97−719.80)	427.81 (285.22−544.34)	n.s.
CRP	13.00 (1.00–30.00)	12.88 (0.30−32.00)	n.s.	20.44 (1.00−145.00)	12.00 (2.00−22.00)	n.s.
CXCL12	1007.10 (599.82–1743.66)	1141.71 (340.77−3026.04)	n.s.	1067.62 (317.83−1823.04)	1016.31 (811.75−1188.45)	n.s.
CXCL13	31.48 (8.27–83.24)	17.69 (3.73−50.68)	n.s.	15.41 (1.89−37.34)	44.29 (3.39−97.34)	n.s.
CXCL5	320.69 (53.44–616.89)	566.66 (252.89−1067.55)	n.s.	598.01 (265.18−1315.25)	425.59 (263.14−653.00)	n.s.
Eotaxin-2	457.93 (385.44–512.80)	506.24 (279.14−655.17)	n.s.	403.04 (85.43−579.55)	408.84 (148.80−591.91)	n.s.
Eotaxin-3	16.04 (10.21–28.20)	39.47 (23.18−61.75)	*	40.68 (9.52−120.52)	88.54 (26.21−195.33)	n.s.
IL-16	24.80 (11.47–55.82)	70.72 (12.83−193.47)	n.s.	47.12 (15.16−113.97)	104.89 (59.74−181.22)	n.s.
IL-20	29.92 (11.49–60.34)	36.54 (0.78−106.11)	n.s.	48.89 (5.52−153.98)	106.79 (24.76−254.13)	n.s.
IL-21	0.91 (0.00–2.25)	27.80 (0.00−70.53)	n.s.	2.73 (0.00−15.76)	19.79 (11.34−26.68)	*
IL-23	129.77 (0.00–283.89)	1923.25 (936.37−3511.30)	*	310.62 (32.49−1086.40)	1409.08 (1202.54−1629.89)	*
IL-28A	8.70 (0.00–25.75)	1240.53 (30.44−3208.29)	*	46.35 (2.35−154.89)	615.55 (28.11−1752.20)	n.s.
IL-33	2.37 (0.00–5.33)	29.47 (0.66−109.11)	n.s.	7.92 (0.00−32.29)	39.47 (10.60−64.37)	n.s.
MCP-2	18.40 (9.47−23.15)	34.33 (23.90−49.68)	*	22.45 (9.89−50.23)	33.28 (19.40−46.30)	n.s.
MCP-4	15.91 (12.19−20.27)	34.44 (18.87−68.22)	n.s.	36.28 (7.87−100.94)	43.84 (25.32−56.12)	n.s.
SCF	13.02 (0.03–38.20)	97.29 (24.81−226.84)	n.s.	15.31 (0.00−44.29)	42.06 (16.12−67.11)	n.s.
TPO	259.73 (197.29−295.80)	1034.02 (517.19−1602.86)	*	372.78 (105.91−936.12)	891.56 (397.49−1556.27)	n.s.
TRAIL	25.52 (15.70−37.82)	64.03 (37.96−86.78)	*	72.06 (21.12−176.74)	63.11 (10.34−134.17)	n.s.
TSLP	1.32 (0.46−2.09)	9.79 (4.04−20.09)	*	2.60 (0.36−9.27)	9.98 (2.31−18.09)	n.s.
creatinine	164.50 (76.00−311.00)	534.67 (140.00−802.00)	n.s.	85.56 (33.00−126.00)	118.00 (90.00−146.00)	n.s.
hemoglobin	12.65 (11.90−13.70)	14.30 (11.50−15.80)	n.s.	12.79 (11.00−15.00)	12.83 (11.30−15.10)	n.s.
leucocytes	10.43 (4.70−17.40)	9.60 (5.70−15.40)	n.s.	6.44 (4.00−11.10)	10.80 (2.00−17.20)	n.s.
thrombocytes	235.50 (170.00−320.00)	253.20 (212.00−348.00)	n.s.	228.62 (148.00−316.00)	298.33 (13.00−591.00)	n.s.

**Table 6:** Cytokines, laboratory parameter, PR3-ANCA titer, and BVAS at active AAV with lung involvement differentiated into patients with and without kidney involvement.

Cytokine	Active (No Lung No Kidney) Mean (min–max); *n* = 12	Inactive (No Lung But Kidney) Mean (min–max); *n* = 6	* = *p* < 0.05; ** = *p* < 0.01; *** = *p* < 0.001; n.s. = not significant
ANCA	317.50 (0.00−2048.00)	266.67 (0.00−1024.00)	n.s.
BVAS	4.92 (1.00−13.00)	13.00 (8.00−19.00)	**
CCL1	3.02 (1.19−8.10)	5.82 (2.35−10.80)	*
CCL15	2489.85 (349.69−3909.46)	3663.79 (322.19−5778.18)	n.s.
CCL17	47.31 (15.50−101.86)	71.40 (0.00−123.75)	n.s.
CCL21	36.44 (0.00−104.05)	76.54 (5.03−256.08)	n.s.
CCL27	365.16 (51.00−719.80)	413.19 (17.98−757.08)	n.s.
CRP	8.94 (0.30−30.00)	14.53 (2.00−32.00)	n.s.
CXCL12	1033.10 (340.77−1743.66)	1286.24 (466.15−3026.04)	n.s.
CXCL13	18.39 (1.89−83.24)	32.43 (3.39−97.34)	n.s.
CXCL5	488.43 (53.44−1067.55)	445.46 (252.89−653.00)	n.s.
Eotaxin-2	405.71 (121.68−579.55)	454.58 (148.80−655.17)	n.s.
Eotaxin-3	27.34 (7.39−69.56)	63.89 (26.21−195.33)	n.s.
IL-16	29.17 (11.47−97.75)	100.36 (36.87 − 193.47)	**
IL-20	30.90 (0.78−95.20)	74.97 (1.92−254.13)	n.s.
IL-21	2.55 (0.00−19.06)	18.13 (0.00−29.46)	*
IL-23	151.86 (0.00−361.18)	1297.14 (936.37−629.89)	***
IL-28A	280.65 (0.00−3208.29)	801.76 (28.11−2838.47)	**
IL-33	2.84 (0.00−12.26)	26.00 (0.86−64.37)	*
MCP-2	21.21 (9.47−50.23)	34.44 (19.40−49.68)	*
MCP-4	24.78 (9.23−43.05)	43.59 (25.32−68.22)	*
SCF	25.84 (0.03−162.36)	70.91 (16.12−226.84)	*
TPO	305.82 (177.72−891.44)	834.78 (397.49−1556.27)	**
TRAIL	58.21 (15.70−176.74)	66.41 (10.34−134.17)	n.s.
TSLP	2.11 (0.46−8.86)	8.32 (2.31−18.09)	**
creatinine	174.45 (33.00−662.00)	294.50 (90.00−802.00)	n.s.
hemoglobin	13.26 (11.80−15.00)	13.93 (11.30−15.80)	n.s.
leucocytes	7.74 (4.00−17.40)	10.90 (2.00−17.20)	n.s.
thrombocytes	222.10 (166.00−320.00)	280.50 (13.00−591.00)	n.s.

## Data Availability

The original data of the cytokine measurements of this study are included within the supplementary information file.

## Supplementary Materials

The following are available online at https://www.mdpi.com/article/10.3390/jcm10122715/s1, Supplementary Figure S1: ANCA titer and CRP do not correlate with disease activity. (A): Correlation of PR3-ANCA titer and BVAS. Correlation was not significant. *n* = 32. (B): Correlation of CRP and BVAS. Correlation was not significant. *n* = 32. Supplementary Table S1. Cytokines, laboratory parameter, PR3-ANCA titer and BVAS at active AAV with and without joint involvement. Supplementary Table S2. Cytokines, laboratory parameter, PR3-ANCA titer and BVAS at active AAV with and without lung involvement.
