# Supplementary material for: Chemokine/Cytokine Levels Correlate with Organ Involvement in PR3-ANCA-Associated Vasculitis
Source: J Clin Med. 2021 Jun 19;10(12):2715. doi: 10.3390/jcm10122715 (PMC8234887; doi:10.3390/jcm10122715)
Supplement: Supplementary file 1 [file jcm-10-02715-s001.zip › jcm-1221008-supplementary.pdf]

Supplementary data

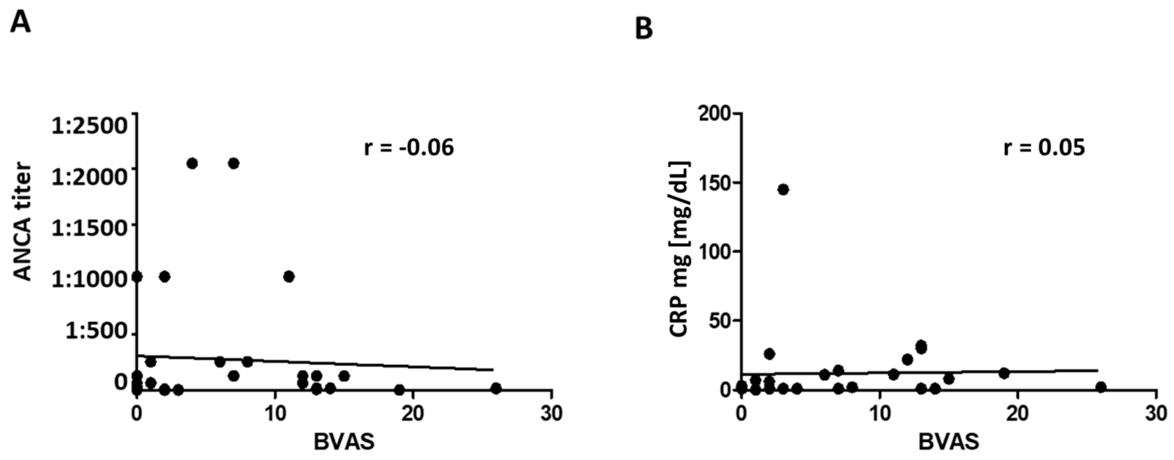

Supplementary Figure S1. ANCA titer and CRP do not correlate with disease activity**. (A)** Correlation of ANCA titer and BVAS. Correlation was not significant. *n* = 32. **(B)** Correlation of CRP and BVAS. Correlation was not significant. *n* = 32.

Supplementary Table S1. Cytokines, laboratory parameter, PR3-ANCA titer and BVAS at active AAV with and without joint involvement.

cytokine	Active (joints affected) mean (min – max); *n* = 9	Inactive (joints and kidney not affected) mean (min – max); *n* = 10	n. s. = not significant
ANCA	422.22 (8.00 – 2048.00)	324.60 (0.00 – 2048.00)	n.s.
BVAS	9.56 (1.00 – 15.00)	6.50 (2.00 – 26.00)	n.s.
CCL1	2.87 (1.19 – 6.39)	4.02 (2.01 – 9.01)	n.s.
CCL15	2594.91 (322.19 – 4627.90)	2327.43 (641.91 – 3909.46)	n.s.
CCL17	39.49 (0.00 – 111.88)	52.83 (23.63 –101.86)	n.s.
CCL21	34.69 (0.00 – 97.60)	62.04 (19.52 – 198.89)	n.s.
CCL27	325.24 (17.98 – 757.08)	380.26 (148.97 – 719.80)	n.s.
CRP	12.94 (0.30 – 32.00)	20.44 (1.00 – 145.00)	n.s.
CXCL12	1081.88 (340.77 – 3026.04)	1067.62 (317.83 – 1823.04)	n.s.
CXCL13	23.82 (3.73 – 83.24)	15.41 (1.89 – 37.34)	n.s.
CXCL5	457.34 (53.44 – 1067.55)	598.01 (265.18 – 1315.25)	n.s.
Eotaxin-2	484.77 (279.14 – 655.17)	403.04 (85.43 – 579.55)	n.s.
Eotaxin-3	29.06 (10.21 – 61.75)	40.68 (9.52 – 120.52)	n.s.
IL-16	50.31 (11.47 – 193.47)	47.12 (15.16 – 113.97)	n.s.
IL-20	33.60 (0.78 –- 106.11)	48.89 (5.52 – 153.98)	n.s.
IL-21	15.85 (0.00 – 70.53)	2.73 (0.00 – 15.76)	n.s.
IL-23	898.41 (0.00 – 3511.30)	310.62 (32.49 – 1086.40)	n.s.
IL-28A	693.05 (0.00 – 3208.29)	46.35 (2.35 – 154.89)	n.s.
IL-33	17.42 (0.00 – 109.11)	7.92 (0.00 – 32.29)	n.s.
MCP-2	27.25 (9.47 – 49.68)	22.45 (9.89 – 50.23)	n.s.
MCP-4	26.21 (12.19 – 68.22)	36.28 (7.87 – 100.94)	n.s.
SCF	59.84 (0.03 – 226.84)	15.31 (0.00 – 44.29)	n.s.
TPO	591.57 (197.29 – 1602.86)	372.78 (105.91 – 936.12)	n.s.
TRAIL	46.91 (15.70 – 86.78)	72.06 (21.12 – 176.74)	n.s.
TSLP	6.02 (0.46 – 20.09)	2.60 (0.36 – 9.27)	n.s.
creatinine	323.14 (76.00 – 802.00)	85.56 (33.00 – 126.00)	n.s.
hemoglobin	13.57 (11.50 – 15.80)	12.79 (11.00 – 15.00)	n.s.
leucocytes	9.97 (4.70 – 17.40)	6.44 (4.00 – 11.10)	n.s.
thrombocytes	245.33 (170.00 – 348.00)	228.62 (148.00 – 316.00)	n.s.

Supplementary Table S2. Cytokines, laboratory parameter, PR3-ANCA titer and BVAS at active AAV with and without lung involvement.

cytokine	Active (lung affected) mean (min - max); $n = 5$	Inactive (lung not affected but kidney) mean (min - max); $n = 6$	* = $p < 0.05$; n.s. = not significant
ANCA	442.40 (4.00 – 2048.00)	266.67 (0.00 – 1024.00)	n.s.
BVAS	11.80 (3.00 – 26.00)	13.00 (8.00 – 19.00)	n.s.
CCL1	4.27 (2.01 – 9.01)	5.82 (2.35 – 10.80)	n.s.
CCL15	2105.68 (641.91 – 3631.09)	3663.79 (322.19 – 5778.18)	n.s.
CCL17	33.53 (14.85 – 50.02)	71.40 (0.00 – 123.75)	n.s.
CCL21	74.40 (19.52 – 198.89)	76.54 (5.03 – 256.08)	n.s.
CCL27	326.88 (148.97 – 526.81)	413.19 (17.98 – 757.08)	n.s.
CRP	37.25 (1.00 – 145.00)	14.53 (2.00 – 32.00)	n.s.
CXCL12	870.22 (317.83 – 1823.04)	1286.24 (466.15 – 3026.04)	n.s.
CXCL13	18.62 (9.02 – 37.34)	32.43 (3.39 – 97.34)	n.s.
CXCL5	629.62 (265.18 – 1315.25)	445.46 (252.89 – 653.00)	n.s.
Eotaxin-2	429.09 (85.43 – 575.17)	454.58 (148.80 – 655.17)	n.s.
Eotaxin-3	45.97 (9.52 – 120.52)	63.89 (26.21 – 195.33)	n.s.
IL-16	60.95 (15.16 – 113.97)	100.36 (36.87 – 193.47)	n.s.
IL-20	61.04 (5.52 – 153.98)	74.97 (1.92 – 254.13)	n.s.
IL-21	17.97 (0.00 – 70.53)	18.13 (0.00 – 29.46)	n.s.
IL-23	1102.01 (32.49 – 3511.30)	1297.14 (936.37 – 1629.89)	n.s.
IL-28A	73.86 (2.35 – 154.89)	801.76 (28.11 – 2838.47)	n.s.
IL-33	33.02 (0.00 – 109.11)	26.00 (0.86 – 64.37)	n.s.
MCP-2	25.24 (12.53 – 39.27)	34.44 (19.40 – 49.68)	n.s.
MCP-4	36.11 (7.87 – 100.94)	43.59 (25.32 – 68.22)	n.s.
SCF	18.47 (0.00 – 44.29)	70.91 (16.12 – 226.84)	n.s.
TPO	653.23 (105.91 – 1602.86)	834.78 (397.49 – 1556.27)	n.s.
TRAIL	58.85 (21.12 – 122.89)	66.41 (10.34 – 134.17)	n.s.
TSLP	7.16 (0.36 – 20.09)	8.32 (2.31 – 18.09)	n.s.
creatinine	71.25 (63.00 – 78.00)	294.50 (90.00 – 802.00)	*
hemoglobin	12.14 (11.00 – 13.50)	13.93 (11.30 – 15.80)	n.s.
leucocytes	7.26 (4.20 – 11.10)	10.90 (2.00 – 17.20)	n.s.
thrombocytes	248.40 (148.00 – 316.00)	280.50 (13.00 – 591.00)	n.s.
